# Nontuberculous mycobacteria – clinical and laboratory diagnosis: experiences from a TB endemic country

**DOI:** 10.2144/fsoa-2020-0023

**Published:** 2020-07-30

**Authors:** Ann Susan Sam, Marilyn Mary Ninan, R Ranjani, Naveen Kumar Devanga Raghupathi, Veeraraghavan Balaji, Joy Sarojini Michael

**Affiliations:** 1 Department of Clinical Microbiology, Christian Medical College, Vellore-632004, India

**Keywords:** geographic distribution, identification, MALDI-TOF mass spectrometry, nontuberculous mycobacteria, prevalence, sequencing, VITEK^®^MS

## Abstract

**Aim:**

To evaluate the performance of VITEK^®^MS with DNA sequencing for laboratory diagnosis of non-tuberculous mycobacteria (NTM) species in a resource-limited setting.

**Methods:**

*16SrRNA* sequencing and MALDI-TOF mass spectrometry (VITEK^®^MS) was performed at a tertiary-care hospital in India. MALDI-TOF results were confirmed by 16S rRNA sequencing. In addition, sequencing of the internal transcribed spacer region was performed on slowly growing NTM.

**Results:**

Commonest species isolated were *M. abscessus, M. intracellulare*, *M. avium, M. fortuitum* and *M. simiae*. *16S rRNA* sequencing and MALDI-TOF results had agreement of 94.5% for rapidly growing and 77.5% for slowly growing NTM.

**Conclusion:**

There is good correlation between VITEK^®^MS and sequencing for rapidly growing NTM. For slowly growing species, sequencing would be required in a third isolates.

The nontuberculous mycobacteria (NTM) are described as *Mycobacteria* other than *Mycobacterium tuberculosis* complex and *Mycobacterium leprae* [1]. There are more than 190 species/subspecies of NTM [2], which were previously considered as environmental organisms of limited clinical relevance. The AIDS epidemic in the 1980s brought a dramatic increase in the numbers of NTM infection, particularly due to those of *Mycobacterium avium* complex. The use of iatrogenic immunosuppression has also added to the burden of disease [3,4]. The spectrum of disease caused by NTM can be classified into four groups – progressive pulmonary disease, cervical lymphadenitis, skin and soft tissue infection and disseminated. Disseminated infections are typically seen only in immunocompromised individuals [5]. Identification of NTM species is extremely important, treatment of these infections are species specific [6].

## Species identification of NTM

Biochemical tests are only useful in the detection of the most common 10–15 species and have a prolonged turnaround time. Hence, newer methods such as high-performance liquid chromatography (HPLC), line probe assay (LPA), DNA probes, matrix-assisted laser desorption ionization-time of flight (MALDI-TOF) and DNA sequencing have been developed. HPLC, LPA and commercial DNA probes have limited utility as they are restricted to identification of the most frequently isolated NTM species. The two MALDI-TOF mass spectrometry (MS) platforms, namely VITEK^®^MS (bioMérieux, Marcy-l'Étoile, France) and Bruker Daltonics platform (Bremen, Germany) for NTM identification, have been evaluated to identify NTM from both solid and liquid media. VITEK^®^MS is a rapid, cost effective and reliable platform, which has the capability to identify as many as 24 NTM species [7,8]. This technology is designed to provide a protein ‘fingerprint’ based on the desorbed ions from cell surfaces by measuring their mass-to-charge ratio. The instrument's software automatically acquires and analyzes the data and generates a profile for comparison with a database of reference spectra, composed of previously well-characterized isolates, providing the species identification of all the common and some of the uncommon NTM species [9].

The latest *in vitro* diagnostic (IVD) database (V3.2.0 Knowledge Base) of VITEK^®^MS allows for better discrimination between species and diversity among strains within the same species [7]. The Spectral Archiving and Microbial Identification System v 4.12 is a specialized research use only library for VITEK^®^MS, containing 1286 spectra from 45 *Mycobacterium* species [10].

The latest version of the Bruker Daltonics platform, also known as MBT Mycobacteria library version 6.0, contains spectra for 178 of the currently known 201 mycobacteria species [11].

Sequencing of the 16S ribosomal RNA gene (rRNA), which is approximately 1500 base pairs, is the most widely used sequencing method for the identification of bacteria and, is considered as the reference standard for identification of NTM species. It identifies both new and rare species, which cannot be identified by existing methods. Region A, at the 5′ end, contains the majority of the species-specific or ‘signature sequences’ in mycobacteria [12]. However, there are many species that cannot be identified by 16S rRNA sequence alone. For example, *M. gastri* and *M. kansasii* sequevars – I & IV – harbor same 16S sequence [13].

Although, another target for DNA sequencing is the internal transcribed spacer (ITS) sequence, which separates the 16S and 23S rRNA genes and is denominated *ITS 1.* The sequence of this fragment comprises of 200–330 base pairs and thus, can easily be analyzed. Several sets of primers that enable the amplification and sequencing of the complete fragment have been published. For the ITS 1 sequence, the high variability utilized for species identification has been documented. Roth *et al.* demonstrated that in situations where 16S rRNA gene sequences were indistinguishable, such as for *M. kansasii* and *M. gastri*, or highly similar, such as for *M. malmoense* and *M. szulgai*, the 16S–23S rRNA gene ITS sequences were a helpful supplement for the differentiation of closely related species. As with the *16S rRNA* gene sequence, *M. marinum* and *M. ulcerans* have identical ITS sequences, thus, cannot be differentiated utilizing this analysis [14,15].

It is important to speciate the NTMs to allow physicians to provide the appropriate treatment and to avoid giving unnecessary treatment for species that has not been associated with clinical significance [6]. The objective of this study was to evaluate the performance of MALDI-TOF by VITEK^®^MS against Sanger sequencing of 16S rRNA and ITS regions.

## Materials & methods

This was a prospective study conducted over a period of 24 months from January 2017 to December 2018 at the Department of Clinical Microbiology, Christian Medical College, (Vellore, India), which is a tertiary care hospital that caters to patients from various parts of the country and from neighboring countries, particularly Bangladesh. 90 NTM culture isolates from pulmonary (39 sputum, 8 bronchoalveolar lavage, 2 lung biopsy and 1 endotracheal aspirate) and extrapulmonary (3 exudates, 9 blood cultures, 4 bone marrow aspirates, 4 tissue biopsies, 3 lymph node biopsies, 3 urine, 2 corneal scraping and 2 catheter tip) samples grown on Lowenstein Jensen medium were included, in accordance to the inclusion and exclusion criteria described below.

### Case definitions

A diagnosis of NTM lung disease was made by isolation of NTM from at least two separate expectorated sputum samples, or a single specimen if it was collected aseptically from a sterile body site such as endotracheal aspirate, bronchoalveolar lavage or lung tissue. To rule out contamination or probable colonization, laboratory results were correlated with the individual's clinical presentation and radiological and histological findings [16].

A case of extrapulmonary NTM infection was defined as having >1 isolate from skin/soft tissue (wound, abscess, tissue or exit catheter); disseminated sites (blood, bone marrow, cerebrospinal fluid, pericardial fluid or peritoneal fluid); lymph node; joint (synovial tissue or joint fluid); or other sites (urine, eye, sinus or nasopharyngeal) [17,18].

### MALDI-TOF

Protein extraction was performed using the VITEK^®^MS Mycobacteria/Nocardia kit. The protocol provided by the manufacturer was followed. Well isolated colonies of NTM from Lowenstein Jensen media were added to a tube with glass beads and ethanol. It was homogenized utilizing a bead beater, followed by inactivation. Formic acid and acetonitrile were added to the lysate. For each organism to be tested, the supernatant was transferred onto the VITEK^®^MS-DS slide spots (bioMerieux, NC, USA). VITEK^®^MS CHCA matrix (α-cyano-4-hydroxycinnamic acid) was added to each sample spot. For instrument calibration, an *Escherichia coli* reference strain (ATCC 8739) was transferred using 1 μl loop to designated spot. Target slides were analyzed by VITEK^®^MS mass spectrometer IVD (version 3.0) database [19].

### Sequencing

Two to three loopfuls (loop of 10 μm diameter) of well-isolated NTM colonies were emulsified in 0.9% saline. DNA extraction was performed utilizing QIAamp^®^ DNA mini kit (Qiagen, Hilden, Germany) with a 1-h preliminary bacterial cell lysis step using lysozyme buffer, followed by DNA quantification using NanoDrop™ 2000 spectrophotometer (Thermo Fisher Scientific, Inc, MA, USA). The extracted product was used for sequencing. The forward and reverse primers used for 16SrRNA sequencing were 5′-AGAGTTTGATCCTGGCTCAG-3′ and 5′-ACGGTTACCTTGTTACGACTT-3′, respectively [20]. The thermal profile of 16S rRNA amplification was as follows: initial denaturation for 15 min at 95°C, followed by 30 cycles of denaturation at 95°C for 1 min, annealing at 52°C for 30 s and extension at 72°C for 1.5 min. The final extension phase was at 72°C for 10 min. The forward and reverse primers used for ITS 1 sequencing were 5′-GTGGGATCGGCGATTGGGAC-3′ and 5′-CCACCATGCGCCCTTAGACAC-3′, respectively [21]. The cycling condition for ITS 1 sequencing was initial denaturation for 95°C for 5 min, followed by 38 cycles of 95°C for 1 min, 55°C for 30 s and 72°C for 1 min and final extension phase of 72°C for 10 min. Sequencing was performed using the ABI PRISM BigDye Terminator Cycle Sequencing kit V3.1 (Applied Biosystems™, CA, USA) and was carried out on the ABI 3500 Genetic Analyzer (Applied Biosystems™). The resulting nucleotide sequences were aligned and Basic Local Analysis Search Tool (BLAST) matched against the reference sequences in the National Center for Biotechnology Information (NCBI; MD, USA) database.

### Data analysis

The clinical and demographic data were analyzed by using the SPSS version 20.0 (IBM, NY, USA) and Microsoft Excel (MS Office 2016) software.

## Results

### Prevalence of NTM

During this 2-year study period, 81,777 samples were received for mycobacterial culture, of which 2775 (3.39%) were culture positive for mycobacteria; among them, 170 (6.12%) were identified as NTM.

### Geographic distribution

The patients included in this study came from different states of India, Bangladesh and Nepal.

### Clinical profiles of the isolates

Fifty of the NTM isolates included in this study (55.5%) were from pulmonary specimens, including sputum, bronchoalveolar lavage, lung biopsy and endotracheal aspirate. 40 isolates were from extrapulmonary specimens such as wound discharges, blood, bone marrow, tissue biopsy, lymph node biopsy, urine, corneal scraping and catheter tip. 49 (54%) were slowly growing NTM and 41 (46%) were rapidly growing NTM. The slowly growing NTM predominantly caused pulmonary infections and disseminated infections in HIV seropositive individuals. Among the 49 patients who presented with NTM pulmonary disease, 40 (81.6%) were due to slowly growing NTM, with the predominant species being identified as *M. intracellulare* (17/40, 42.5%). Twenty five patients with pulmonary disease had past history of tuberculosis (25/50 = 50%) and 20 of them had associated bronchiectasis (20/50 = 40%).

Disseminated infection was the second most common presentation of slowly growing NTM (7/90, 7.77%). *M. avium*, *M*. *intracellulare* and *M. simiae* were isolated from bone marrow and lymph node specimens. Six out of these seven patients had AIDS, with a CD_4_ cell count less than 150 cells/μl at the time of diagnosis. The median CD_4_ count of these patients was 124 cells/μl. One of the patients who presented with disseminated disease was a 3-year-old child with severe combined immunodeficiency disorder (SCID) from whom *M. avium* was isolated from tissue biopsy of the middle ear.

However, rapidly growing NTM's produced a wider spectrum of infections – particularly skin and soft tissue infections – with postoperative wound infections being the most common (Table 2).

**Table 1.** Geographic distribution of the study population whose isolates were included in the study.

Geographic area	Number of cases, percentage
Tamil Nadu, India	25/90, 28%
West Bengal, India	23/90, 23%
Jharkhand, India	12/90, 13%
Andhra Pradesh, India	10/90, 11
Kerala, India	6/90, 7%
Bangladesh	5/90, 6%
Bihar, India	4/90, 4%
Chhattisgarh, India	2/90, 2%
Karnataka, India	2/90, 2%
Nepal	1/90, 1%

**Table 2.** Clinical presentations of rapidly growing nontuberculous mycobacteria.

Clinical presentation	Number of cases, percentage
Skin and soft tissue infections	16/41, 39
Blood stream/line associated infections	9/41, 21.9
Pulmonary infections	9/41, 21.9
Injection site abscesses	2/41, 4.8
Infections of the urinary system	2/41, 4.8
Breast abscess	1/41, 2.4
Skull base osteomyelitis	1/41, 2.4
Chemotherapy port site infection	1/41, 2.4

### Species identification by sequencing

Sequencing of the 16S rRNA (reference standard) was used to confirm the species identification obtained by MALDI-TOF. The most common slowly growing NTMs isolated were *M. intracellulare* (19 isolates) followed by *M. simiae* (8 isolates)*, M. avium* (6 isolates), *M. avium* complex (3 isolates), *M. kansasii* (2 isolates), *M. europaeum* (2 isolates), *M. szulgai* (1 isolate)*, M. yongonense* (1 isolate)*, M. timonense* (1 isolate)*, M. scrofulaceum* (1 isolate)*, M. parascrofulaceum* (1 isolate) and *M. longobardum* (1 isolate). Species with intermediate growth rate such as *M. flavescens* (2 isolates) and *M. novocastrense* (1 isolate) have been included along with slowly growing NTM's in this study. The most common rapidly growing NTM isolated were *M. abscessus* (26 isolates) followed by *M. fortuitum* (10 isolates)*, M. farcinogenes* (1 isolate)*, M. bacteremicum* (1 isolate)*, M. mucogenicum* (1 isolate), *M. phocaicum* (1 isolate) and *M. goodii* (1 isolate).

ITS sequencing was performed on all the slowly growing NTM. Out of the six *Mycobacterium avium* isolates, all were identified as *M. avium* subspecies *hominisuis* by ITS sequencing. The results were compared with 16S rRNA sequencing and MALDI-TOF as described in the Table 3. 16S rRNA and ITS sequencing had an agreement of 95.5% (95% CI: 82.4–99.7%). MALDI-TOF and ITS sequencing of slowly growing NTM demonstrated 76% agreement (95% CI: 63.9–81.4%).

**Table 3.** Species identification of nontuberculous mycobacteria.

Species-based on 16S rRNA sequencing	ITS sequencing	MALDI-TOF ID
	Number of concordant isolates	Number of concordant isolates
*M. intracellulare (n = 19)*	19	14 (5 were not identified)
*M. simiae (n = 8)*	8	8
*M. avium (n = 6)*	6	5 (1 was not identified)
M. avium complex *(n = 3)*	3 (identified as *M. intracellulare*)	2 (1 was not identified)
*M. kansasii (n = 2)*	2	2
*M. flavescens (n = 2)*	2	2
*M. europaeum (n = 2)*	2	0 (1 was identified as *M. gordonae*, 1 was not identified as it was not included in the database)
*M. szulgai (n = 1)*	1	1
*M. yongonense (n = 1)*	1	Not identified as it was not included in the database
*M. timonense (n = 1)*	1	0 (*M. avium* complex)
*M. novocastrense (n = 1)*	0 (*M. flavescens*)	0 (*M. flavescens*)
*M. scrofulaceum (n = 1)*	0 (*M. parascrofulaceum*)	0 (no identification)
*M. parascrofulaceum (n = 1)*	1	Not included in the database
*M. longobardum (n = 1)*	0 (*M. celatum*)	Not included in the database
*M. abscessus* (*n* = 26)	^†^	24 (2 were not identified)
*M. fortuitum* (*n* = 10)	^†^	10 (*M. fortuitum* group)
*M. farcinogenes (n* = 1)	^†^	1
*M. bacteremicum (n* = 1)	^†^	0 (*M. neoaurum*)
*M. mucogenicum* (*n* = 1)	^†^	1
*M. phocaicum* (*n* = 1)	^†^	0 (*Pseudomonas stutzeri*)
*M. goodii* (n = 1)	^†^	Not included in the database

† = not performed; results of discordant isolates given in the parenthesis.

ITS: Internal transcribed spacer; MALTI-TOF: Matrix-assisted laser desorption ionization-time of flight.

### Species identification by matrix-assisted laser desorption ionization mass spectrometry

MALDI-TOF using VITEK^®^MS was evaluated against the aforementioned DNA sequencing methods. MALDI-TOF identified 76 of the 90 isolates up to species or complex/group level. However, it could not identify 12 (12/90, 13.3%) isolates. Two isolates were misidentified as non-mycobacterial species, in other words, *M. goodii* as *Staphylococcus equorum* and *M. phocaicum* as *Pseudomonas stutzeri*). Species that were not identified by MALDI-TOF included *M. intracellulare*, *M. abscessus*, *M. avium* complex, *M. yongonense, M. longobardum* and *M. scrofulaceum* (Table 3).

The statistical agreement between the species identification by MALDI-TOF, with the reference standard 16S rRNA sequencing, is described in Table 4.

**Table 4.** Agreement between the results of 16S rRNA sequencing, ITS sequencing and MALDI-TOF.

Tests compared (Total tests, n = 90)	Statistical agreement	95% CI
Concordance of 16S rRNA sequencing and MALDI-TOF • Rapidly growing NTM • Slowly growing NTM	83% (cumulative) 94.5% 77.5%	74.00–90.4% 81.3–99.4% 63.9–81.4%
Concordance of ITS sequencing for slowly growing NTM and MALDI-TOF	76%	63.9–81.4%

ITS: Internal transcribed spacer; MALDI-TOF: Matrix assisted laser desorption ionization-time of flight; NTM: Non-tuberculous mycobacteria.

## Discussion

According to the American Thoracic Society (ATS; NY, USA)/Infectious Diseases Society of America (IDSA; VA, USA) statement of 2007, “*clinically significant NTM isolates should be routinely identified to the species level*” [16]. The British Thoracic Society (London, UK) guidelines in 2017 also recommends that NTM should be identified to the species level utilizing validated molecular or mass spectrometry techniques, as treatment of NTM infections are species-specific and therefore, pathogens must be distinguished from environmental contaminants [6]. Thus, timely and accurate identification of NTM is required to guide therapy.

### Prevalence of NTM

A study on the retrospective analysis of the isolation rates of NTM from various clinical specimens was conducted at this institute during the period of 1999–2004 [22]. The prevalence of NTM at that time period was 3.9%. The current study demonstrated the prevalence of NTM as 6.12%, which corresponds to the increase in the numbers of NTM globally. However, data from the South Korean national healthcare insurance data identified the age-adjusted prevalence as 33.3 cases/100,000 population during 2003–2016 [23]. The lack of a nation-wide registry for NTM cases in India is probably reflected in the lower prevalence rate noted in this study.

### Geographic distribution

The study was performed at a tertiary referral center in South India. The study population was from the Indian states and neighboring countries as shown in Table 1. The climatic conditions of Indian states vary widely and the relationship between climate and the occurrence of NTM infections has not been studied across the country to date. However, NTM is ubiquitously present in the environment and can colonize the human epithelia, which could lead to opportunistic infections, particularly wound infections if the healthcare facilities are inadequate [24–26].

### Role of other lung diseases in the occurrence of NTM

We observed that the pulmonary NTM infections often occurred in the individuals with a history of tuberculosis and bronchiectasis, although it is well established that lung damage due to previous tuberculosis is a risk factor for NTM pulmonary disease [27].

### NTM infections & associated immunodeficient states

Of the seven patients with disseminated NTM infection, six were diagnosed with AIDS, with a median CD_4_ count of 124 cells/cu.mm, which was higher than the other previously reported studies conducted in the patients with disseminated NTM infections, which determined a median CD4 count of 10 cells/cu.mm. This may be due to the higher prevalence of NTM in our environment and the healthcare setting [28–30].

### Spectrum of clinical features

The most common clinical manifestation of patients with rapidly growing NTM were skin and soft tissue infections – predominantly due to postoperative wound infections. NTM bacteremia with rapidly growing NTM was seen in seven patients with prolonged hospitalization of more than 2 weeks, who had central venous line *in situ*, had undergone invasive procedures of the cardiovascular system (stent, valve repair) or were undergoing therapy for hematological malignancies or immune system disorders. This finding correlates with the studies from other centers in USA [31].

### Species-wise distribution

*M. abscessus* was the most common species identified during the study period, followed by *M. intracellulare* (Table 3). We observed that there were no *M. chelonae* isolates identified during the study period, which is in contrast with a previous study conducted in this hospital, which identified 46% NTM isolates as *M. chelonae* [22]. The number of *M. simiae* cases are also on the rise in the study population. The NTM-Network European Trials Group (NET; Borstel, Germany) conducted a multicentric study in 2008 to find the relative distribution of the different NTMs in pulmonary samples. *Mycobacterium avium* complex (MAC) predominated in most countries (47%), followed by *M. gordonae* (11%). *M. xenopi* and *M. gordonae* had a high rate of isolation in Europe. Isolates from Asia (Japan, South Korea and Taiwan) were predominantly MAC (54%) followed by a significant proportion of rapid growers (31%) [32]. In contrast to this data, a study from North India by Umrao *et al*. determined that the slowly growing NTMs were isolated more frequently (59%) when compared with rapidly growing species (41% of isolates) [33].

### Molecular methods & NTM species identification

In this study, complete sequencing of the 16S rRNA gene of 90 NTM isolates was performed. A percentage similarity of 99–99.9% or more was regarded as the cut-off for the identification of the most accurate species as per the CLSI MM18-A, 2008 guidelines [34]. The most common species identified were *M. abscessus, M. intracellulare, M. fortuitum. M. simiae* and *M. avium*, which is similar to the studies from around the globe. However, in a high burden low resource country like India, complete 16S rRNA sequencing can only be performed at referral laboratories and is not cost effective to be performed on every isolate.

Three isolates, which were identified as *M. avium* complex by 16S rRNA sequencing, were found to be *M. intracellulare* on ITS sequencing. The ITS 1 sequence has a high variability and can be used for species discrimination, especially with slowly growing species including MAC [15]. In this study, ITS sequencing was performed on all slowly growing NTM isolates. All the MAC isolates were identified up to the species level, while *M. avium* was identified up to subspecies level. Out of the 6 *M. avium* isolates, all were identified as *M. avium* subspecies *hominisuis*, which is the most pathogenic subspecies to humans according to various studies [35]. Rare species such as *M. yongonense* and *M. timonense* can be identified using this method. Hence, ITS sequencing can be utilized as an additional method over 16S rRNA sequencing for identification of slowly growing, as well as novel/rare NTM species.

### MALDI-TOF MS & NTM species identification

The accuracy of this method is dependent upon obtaining good quality spectra. This can be challenging due to the complex cell wall of Mycobacteria which needs specialized protein extraction kits.

In this study, isolates were tested using the VITEK*^®^*MS system, and the results were analyzed using the VITEK*^®^*MS IVD version 3.0 database. As the results have demonstrated, there are gaps to be filled in the accurate species-level identification of NTM. 13 out of the 90 isolates (14.4%) were not given any genus or species-level identification and 2 were misidentified (2/90, 2%) as nonmycobacterial species. Of the 13 isolates that were not identified, 3 species were not in the database, namely *M. europaeum*, *M. yongonense* and *M. longobardum*. However, some of the *M. intracellulare* (5 out of 19 isolates)*, M. abscessus* (2 out of 28 isolates), MAC, *M. scrofulaceum* and *M. avium* isolates were not identified even though they were included in the database. *M. timonense, M. parascrofulaceum, M. bacteremicum and M. farcinogenes* were identified as *M. intracellulare*, *M. scrofulaceum*, *M. neoaurum* and *M. fortuitum* group, respectively. The studies that evaluated the MALDI TOF MS platforms for the NTM identification also highlighted similar results – for instance 7/244 [3%] and 2/244 [1%] isolates were misidentified by the Biotyper system and the VITEK^®^MS system, respectively [36].

Although MALDI-TOF by VITEK^®^MS has been successful in the identification of many species of NTM, it cannot distinguish several clinically significant species and subspecies, including *M. abscessus* subsp. *abscessus*, *M. abscessus* subsp. *massiliense* and *M. abscessus* subspecies, *M. bolletii*; *M. chelonae* and *M. abscessus*; *M. intracellulare* and *M. chimaera*; *M. mucogenicum* and *M. phocaicum;* subspecies of *M. avium* and *M. abscessus;* and species within the *M. fortuitum* group. In spite of good quality spectrum, if there is a ‘no identification’ call, this may indicate that the organism is not represented in the database of the MALDI-TOF MS platform. Newer species are added and databases are regularly updated by manufacturers. In contrast, if the spectral pattern is poor and multiple species identification are given, there may be mixed culture present, and purity of the isolate should be confirmed [36]. In a similar study performed by Luo *et al.* on the evaluation of VITEK^®^MS version 3.0 for NTM identification, 425/507 (83.8%) isolates were identified the first time, 51/507 (11.24%) additional isolates were identified on repeat, 23/507 (4.53%) isolates remained unidentified and 8/507 (1.6%) isolates were misidentified at species level. Among slowly growing NTM, only 2 out of 56 (3.6%) *M. avium* and 3 out of 153 (2%) *M. intracellulare* were unidentified, which was marginally better than the findings in this study [8]. The concordance of 77.5% between VITEK^®^MS and 16S rRNA sequencing in the identification of slowly growing Mycobacteria is predominantly due to the fact that MALDI-TOF requires moderate growth to be present on the solid media for identification, rather than the scant growth required for sequencing, as demonstrated by other studies as well [37]. Therefore, MALDI-TOF needs to be performed initially for all SGMs as it is cost effective and, in those isolates, which have no identification, sequencing will be required.

### MALDI-TOF platforms & methodologies

There have been various studies in the past few years that have evaluated the various platforms and databases for the species identification of NTM. Overall performance in the identification of NTM clinical isolates utilizing MALDI-TOF MS was comparable between the bioMérieux VITEK^®^MS and Bruker MALDI Biotyper systems (MA, USA) in a study by Brown-Elliott *et al.* [36]. The systems identified 92% (225/244) and 95% (231/244) of all isolates to at least the complex/group level, respectively. The Biotyper system identified 151/244 isolates (62%) to the highest taxonomic level, either the species or subspecies level, depending on the organism. The VITEK^®^MS system identified 138/244 of all isolates (57%) to the highest taxonomic level. The limitations common to both platforms is that they cannot differentiate among members of *M. abscessus* complex, *M*. *fortuitum* group, *M. avium* complex and *M mucogenicum* group. This is evident from this study as well. Both platforms cannot identify *M. terrae* reliably. However, this could not be assessed as there were no *M. terrae* isolates identified by sequencing in this study.

The latest database for clinical use in the VITEK ^®^MS platform, in other words, IVD v. 3.2.0 and the database used in this study (IVD 3.0) has no difference in the species included [19,38].

MALDI-TOF is less expensive per test, after the initial purchase of the mass spectrometry instrument, which makes it a suitable assay for routine diagnostics. Sequencing is five- to six-times more expensive than MALDI-TOF and therefore, should be used as a confirmatory test. MALDI-TOF requires only protein extraction, which is a simple technique that can be performed by any laboratory technologist in a routine clinical microbiology laboratory setting. However, sequencing needs trained personnel for the procedure in a molecular laboratory setting. MALDI-TOF is a rapid method that takes 1.5 to 2 h for the whole procedure, whereas sanger sequencing takes on average 12–24 h [39].

## Limitations

The emended genus *Mycobacterium* encompasses the ‘*Tuberculosis-Simiae*’ clade, and four novel genera viz. *Mycolicibacterium* gen. nov., *Mycolicibacter* gen. nov., *Mycolicibacillus* gen. nov. and *Mycobacteroides* gen. nov. corresponding to the ‘*Fortuitum-Vaccae*’, ‘*Terrae*’, ‘*Triviale*’ and ‘*Abscessus-Chelonae*’ clades, respectively. However, the isolates included in this study have continued to use the previous classification as the emended genus names were not available in the VITEK^®^MS database at the time of study.

The subspecies of MABC (*Mycobacterium abscessus* complex) differs in their antibiotic susceptibility profiles and disease prognosis. Subspecies-level identification of *M. abscessus* could not be performed using the sequencing of 16S rRNA or MALDI-TOF by VITEK^®^MS.

Climatic conditions of the geographic area from which the patients come from could not be addressed in this study.

## Conclusion

MALDI-TOF is a relatively novel technology, especially in a resource-limited country. It is an excellent method for diagnostic stewardship for species identification of rapidly growing NTM, as it is simple-to-use, rapid, less expensive (after the initial purchase of the mass spectrometry instrument) and accurate. Targeted gene sequencing may be required for the rare and novel species of NTM, particularly the slowly growing species as the database lacks the rare species. However, gene sequencing needs to be performed using multiple targets for complete identification up to subspecies level, which may be available only in the reference laboratories.

## Future perspective

As infections due to NTM are on the rise, there is a necessity for timely diagnosis and accurate speciation for appropriate patient management. This study has demonstrated that MALDI-TOF mass spectrometry is an essential tool to be integrated into routine clinical microbiology laboratories and DNA sequencing technology in diagnostic laboratories with adequate infrastructure.

**Executive summary**

- The prevalence of nontuberculous mycobacteria (NTM) during the study period was 6.12%, which is in agreement with the increase in the numbers of NTM globally.
- The cumulative concordance of MALDI-TOF with the reference standard 16S rRNA sequencing was 83.33%.
- MALDI-TOF assay is a rapid and cost-effective method for identification of NTM in a routine diagnostic laboratory, particularly for the rapidly growing species.
- Clinical profile of these isolates was also studied which showed pulmonary infection (55%) being the most common clinical presentation of slowly growing species and skin and soft tissue infection (56%) for the rapidly growing species.
- The numbers of invasive infections due to NTM such as *Mycobacterium abscessus* is on the rise.
- MALDI-TOF is useful for species identification of rapidly growing NTM as it is simple-to-use, rapid, less expensive and accurate.
- VITEK^®^MS cannot distinguish several clinically significant species and subspecies, including *M. abscessus* subsp. *abscessus*, *M. abscessus* subsp. *massiliense* and *M. abscessus* subsp. *bolletii*; *M. chelonae* and *M. abscessus*; *M. intracellulare* and *M. chimaera*; *M. mucogenicum* and *M. phocaicum;* subspecies of *M. avium* and *M. abscessus;* and species within the *M. fortuitum* group.
- Targeted gene sequencing may be required for the rare and novel species of NTM, particularly the slowly growing species as the database lacks the rare species.
